# Donepezil for Dementia with Lewy Bodies: A Randomized, Placebo-Controlled Trial

**DOI:** 10.1002/ana.23557

**Published:** 2012-07-23

**Authors:** Etsuro Mori, Manabu Ikeda, Kenji Kosaka, on behalf of the Donepezil-DLB Study Investigators

**Affiliations:** 1 Department of Behavioral Neurology and Cognitive NeuroscienceTohoku University Graduate School of Medicine, Sendai; 2 Department of Psychiatry and Neuropathobiology, Faculty of Life SciencesKumamoto University, Kumamoto; 3 Department of Psychiatry, Yokohama City University School of MedicineYokohama, Japan

## Abstract

**Objective:** Because cholinergic deficits are prominent in dementia with Lewy bodies (DLB), we investigated the effects of a cholinesterase inhibitor, donepezil, in such patients in a randomized, double-bilnd- placebo-controlled exploratory phase 2 trial.

**Methods:** One-hundred forty patients with DLB, recruited from 48 specialty centers in Japan, were randomly assigned to receive placebo or 3, 5, or 10mg of donepezil hydrochloride daily for 12 weeks (n = 35, 35, 33, and 37, respectively). Effects on cognitive function were assessed using the Mini-Mental State Examination (MMSE) and several domain-specific neuropsychological tests. Changes in behavior were evaluated using the Neuropsychiatric Inventory, caregiver burden using the Zarit Caregiver Burden Interview, and global function using the Clinician’s Interview-Based Impression of Change-plus Caregiver Input (CIBIC-plus). Safety measures included the Unified Parkinson’s Disease Rating Scale part III.

**Results:** Donepezil at 5 and 10mg/day was significantly superior to placebo on both the MMSE (5mg: mean difference, 3.8; 95% confidence interval [CI], 2.3-5.3; *p* < 0.001; 10 mg: mean difference, 2.4; 95% CI, 0.9-3.9; *p* = 0.001) and CIBIC-plus (*p* < 0.001 for each); 3mg/day was significantly superior to placebo on CIBIC-plus (*p* < 0.001), but not on the MMSE (*p* = 0.017). Significant improvements were found also in behavioral measures (*p* < 0.001) at 5 and 10mg/day and caregiver burden (*p* = 0.004) at 10 mg/day. The safety results were consistent with the known profile of donepezil and similar among groups.

**Interpretation:** Donepezil at 5 and 10mg/day produces significant cognitive, behavioral, and global improvements that last at least 12 weeks in DLB patients, reducing caregiver burden at the highest dose. Donepezil is safe and well tolerated.

## INTRODUCTION

Dementia with Lewy bodies (DLB) is a common form of dementia in the elderly, and constitutes the second largest group of patients with dementia after Alzheimer disease (AD).^1^ The core clinical features of DLB include neuropsychiatric symptoms and motor symptoms of parkinsonism as well as cognitive impairment characterized by deficits of attention, executive function, and visual perception.^2^ Fluctuating cognition, hallucinations, and delusions are major sources of difficulties and distress for both patients and caregivers. The motor and autonomic features further impair activities of daily living and lead to poorer quality of life.^3^, ^4^ However, pharmacological management of DLB remains challenging, because it is complicated by the risk of adverse reactions to medication.^5^ Treatments for one aspect of the disease may exacerbate other symptoms. It is well recognized that DLB patients can be exquisitely sensitive to antipsychotic agents and can develop life-threatening sensitivity reactions.^6^ Antiparkinson medication given to improve motor symptoms can exacerbate neuropsychiatric symptoms such as hallucinations. There are no approved treatments for DLB.

Cholinergic loss in DLB is associated with deficits in attention and cognition, and also with neuropsychiatric symptoms.^7^ Neuropathological and neuroimaging studies have demonstrated that cholinergic neurotransmission is more defective in DLB than in AD.^8^ Although cholinergic losses in DLB affect both brainstem and basal forebrain presynaptic nuclei, in contrast to AD, postsynaptic cortical muscarinic and nicotinic receptors are more functionally intact,^9^ suggesting that cholinesterase inhibitors (ChEIs) may be potent for DLB. Case reports and open-label studies have demonstrated the benefit of galantamine, rivastigmine, and donepezil on cognitive and behavioral symptoms in DLB.^10-14^ However, only 1 randomized placebo-controlled trial (RCT) has been reported, in which it was suggested that rivastigmine improved attentional and behavioral symptoms.^15^ Memantine, an N-methyl-D-aspartate receptor antagonist, was also tested in 2 RCTs including DLB patients; however, the results were equivocal.^16^, ^17^ Therefore, there is very little evidence for pharmacotherapy in this group. The aim of this phase 2 study was to exploratively investigate the efficacy and safety of donepezil hydrochloride, in 3 different doses compared to placebo, in patients with DLB. This study was registered as number NCT00543855.

## Patients and Methods

### Patients

Patients who met the consensus diagnostic criteria^2^ for probable DLB were recruited from 48 psychiatric or neurological specialty centers throughout Japan from October 2007 to February 2010. Diagnosis of each patient was validated after discussion by the central committee. Outpatients (≥50 years old) with mild to moderate-severe dementia (10-26 on the Mini-Mental State Examination [MMSE]^18^ and Clinical Dementia Rating^19^ ≥0.5) and with behavioral symptoms (Neuropsychiatric Inventory-plus [NPI-plus] ≥8) were eligible. NPI-plus is a 12-item version of the NPI, with the original 10 items supplemented by 2 DLB-relevant domains of sleep and cognitive fluctuation.^11^, ^20^, ^21^ Patients had caregivers who routinely stayed with them at least 3 days per week and 4 hours per day, provided information for this study, assisted compliance with treatment, and escorted patients to required visits.

Exclusion criteria included Parkinson disease diagnosed at least 1 year prior to the onset of dementia; focal vascular lesions on magnetic resonance imaging or computed tomography that might cause cognitive impairment; other neurological or psychiatric diseases; clinically significant systemic disease; complications or history of severe gastrointestinal ulcer, severe asthma, or obstructive pulmonary disease; systolic hypotension (<90mmHg); bradycardia (<50m^−1^); sick sinus syndrome; atrial or atrioventricular conduction block; QT interval prolongation (≥450 milliseconds); hypersensitivity to donepezil or piperidine derivatives; severe parkinsonism (Hoehn and Yahr score ≥ IV)^22^; and treatment with ChEIs or any investigational drug within 3 months prior to screening. ChEIs, antipsychotic agents, and antiparkinson drugs other than L-dopa or dopamine agonists were not allowed during the study.

Written informed consent was obtained from the patient (if at all possible) and his/her caregiver before initiating the study procedures. The study was conducted in accordance with the principles of the Declaration of Helsinki. The protocol was approved by the institutional review board at each center.

### Randomization and Masking

This was a multicenter, randomized, double-blind, parallel-group, placebo-controlled study. Treatment lasted 14 weeks, including a 2-week prerandomization period followed by a 12-week randomization period. All participants were given placebo tablets during the prerandomization period, after which the patients were randomly assigned in a 1:1:1:1 ratio to placebo, 3, 5, or 10mg of donepezil. The randomization list was computer-generated using a randomized block design to allocate several blocks (size 4) to each center. Patients were sequentially assigned to the lowest randomization number available at the time of each enrollment at each center. The randomization list was securely managed by an allocation officer, who was independent of all parties concerned with the study, at Bellsystem24, Tokyo, Japan, until study completion. Access to the list was not allowed except in emergency. Study personnel and participants were unaware of the treatment assignment. During the study period, the code was broken for 2 patients: 1 each in the placebo and 3mg arms because of serious adverse events (pelvic fracture and subarachnoid hemorrhage, respectively).

Patients received 2 study drug tablets, which were composed of a combination of 3mg, 5mg, or matched placebo tablets with the same physical appearance, once daily in the morning. Dosage was titrated at the beginning of the randomization period in the 5 and 10mg groups. In the 5mg group, treatment began with 3mg for 2 weeks, and then the dose was increased to 5mg. The 10mg group started with 3mg for 2 weeks, followed by 5mg for 4 weeks, after which the 10mg dose was provided for 6 weeks. The dose was escalated after patient safety was confirmed by telephone interview.

### Procedures

This study had no formal predefined primary endpoint. However, cognition, behavior, global function, and caregiver burden were determined as core efficacy outcomes prior to the study initiation.

Efficacy was assessed at baseline and weeks 4, 8, and 12. Cognition was assessed using the MMSE.^18^ In addition, 3 cognitive domains (attentive, executive, and visuoperceptual functions) relevant to DLB were assessed using the Wechsler Memory Scale-Revised (WMS-R) attention/concentration subscale,^23^ the Verbal Fluency test (category and letter),^24^ the Wechsler Adult Intelligence Scale (WAIS-III) symbol digit modalities subscale,^25^ and the Visual Perception Test for Agnosia form discrimination and overlapping figure identification subscales.^26^

Behavior was assessed using the NPI-plus (the original NPI-10 consisting of 10 behavioral domains; ie, delusions, hallucinations, agitation/aggression, dysphoria, anxiety, euphoria, apathy, disinhibition, irritability/lability, and aberrant motor behavior, supplemented by 2 DLB-relevant domains of sleep and cognitive fluctuation).^11^, ^20^, ^21^ The questions in the cognitive fluctuation domain were arranged according to those reported in the literature.^27^, ^28^ In addition to the NPI-10 and each domain of the NPI-plus, a 2-item subscore (NPI-2) was calculated as the sum of scores for hallucinations and cognitive fluctuation, which correspond to the core features of DLB in the consensus criteria, and a 4-item subscore (NPI-4) calculated as the sum of scores for delusions, hallucinations, apathy, and depression, which were reported as the main DLB symptom cluster in the previous rivastigmine study.^15^

Changes in global clinical status were assessed by an experienced clinician who was not involved in patient management or other assessments, using the Clinician’s Interview-Based Impression of Change plus Caregiver Input (CIBIC-plus), with 7 grades ranging from Marked Improvement to Marked Worsening.^29^

Caregiver burden was assessed using the Zarit Caregiver Burden Interview (ZBI), which evaluates the physical, psychological, and social consequences of caring activities.^30^ The ZBI contains 22 items scored from 0 (best) to 4 (worst), from which a total score from 0 to 88 is calculated.

Motor function was assessed as a safety measure using the Unified Parkinson’s Disease Rating Scale (UPDRS) part III at baseline and week 12.^31^ Safety was also assessed on the basis of adverse events (AEs), vital signs, electrocardiogram, and laboratory tests.

### Statistical Analyses

The sample size was originally defined as 160 patients (40 in each arm) on the basis of feasibility considerations. However, because of recruitment difficulties, the sample size was reduced to 140. Although no formal calculation of power was performed, this number of patients should have provided a roughly 80% power to detect a 40% difference in responder rates (see definition below) between the active and placebo arms with a 2-sided significance level of 0.0167.

The safety analysis set comprised all patients who received at least 1 dose and had a postbaseline safety assessment. Efficacy analyses were performed on the full analysis set, which consisted of all patients who had at least 1 valid postbaseline assessment on any of the efficacy scales, with the last observation carried forward (LOCF).

Imbalances in baseline demographics and background characteristics were assessed by analysis of variance (ANOVA), Kruskal-Wallis test, or chi-square test, with a 2-sided significance level of 0.15.

For efficacy, mean changes from the baseline in each outcome measure other than CIBIC-plus were compared between each active group and placebo by both Student *t* test and analysis of covariance (ANCOVA), with baseline values (sex, weight, and each test score) as covariates. In addition to the LOCF analysis, the mixed-effect model for repeated measures was applied to analyze data with dropouts as the secondary approach. For CIBIC-plus, the Wilcoxon rank sum test was used to compare the grade distributions between each active group and placebo. In addition, the MMSE and CIBIC-plus were analyzed by number of responders, defined as a ≥3 point improvement on the MMSE and as -minimal improvement- or better on the CIBIC-plus. Fisher exact probability test was used to compare each active group to placebo. The dose-response relationship (linear or 5mg saturation) across the 3 doses was also analyzed, as a secondary analysis, by ANOVA with contrasts for the MMSE and NPI, and by Cochran-Armitage test with contrasts for the CIBIC-plus. Significance levels were set at 2-sided 0.0167 for comparison with placebo (multiplicity adjustment by Bonferroni method) and 2-sided 0.05 for trend analysis.

The incidence of AEs was calculated, and group differences were examined by Fisher exact probability test. For laboratory parameters and vital signs, descriptive statistics and frequency distributions were calculated. UPDRS part III scores were compared between each active group and placebo by both Student *t* test and ANCOVA, with baseline values as covariates. Significance level was set at 2-sided 0.05 for safety analysis.

All analyses were made on SAS version 9.1.3 (SAS Institute, Cary, NC).

## Results

### Patients

**Fig. 1:**
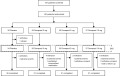
Patient disposition. SAS = safety analysis set.

Of the 167 patients screened in the prerandomization period, 140 were randomized to the 4 groups (35, 35, 33, and 37 to placebo, 3mg, 5mg, and 10mg, respectively). In the randomization period, 1 patient in the placebo group was withdrawn before receiving the study drug due to refusal, and was excluded from the safety analysis set. Two patients (1 each in the placebo and 10mg groups) who did not meet probable DLB criteria and 2 patients (1 each in the placebo and 5mg groups) with no postbaseline efficacy assessments were excluded from the full analysis set. Thus, the safety analysis set included 139 patients (34, 35, 33, and 37 in the placebo, 3mg, 5mg, and 10mg groups, respectively), and full analysis set included 135 patients (32, 35, 32, and 36, respectively). Sixteen patients (4, 4, 2, and 6, respectively) withdrew during the study; LOCF was applied to them (Fig 1). In the 10mg group, 3 patients discontinued during the titration phase: 1 at the 3mg/day and 2 at the 5mg/day period.

Baseline patient characteristics are summarized in Table 1. The 4 groups were similar for demographic and disease-related characteristics, although significant differences were found in the distributions of women and men, and mean body weight. Because women predominated, mean body weight was lower in the 10mg group. Some patients were taking L-dopa, dopamine agonists, antidepressants, or benzodiazepines at baseline. However, the proportion of each medication was not different among groups. Baseline scores of the cognitive and behavioral scales were also comparable, although the mean NPI-10 score was lower in the 5mg group, and the WAIS-III symbol digit score was lower in the placebo group (Table 2).

**Table 1:** Baseline Characteristics of Patients (Full Analysis Set)

Characteristic	Treatment Group				*p*
	Placebo, n = 32	Donepezil			
		3mg, n = 35	5mg, n = 32	10mg, n = 36	
Age, yr	78.6 (4.7)	79.6 (4.5)	77.9 (6.8)	78.6 (6.1)	0.663
Sex					0.001
Male	9 (28.1%)	17 (48.6%)	16 (50.0%)	4 (11.1%)	
Female	23 (71.9%)	18 (51.4%)	16 (50.0%)	32 (88.9%)
Weight, kg	47.5 (9.0)	51.3 (10.1)	49.6 (9.6)	44.9 (9.2)	0.035
CDR					0.643
0	0	(0.0%)	0 (0.0%)	0 (0.0%)	0 (0.0%)	
0.5	8 (25.0%)	10 (28.6%)	12 (37.5%)	9 (25.0%)	
1	20 (62.5%)	18 (51.4%)	16 (50.0%)	20 (55.6%)	
2	3 (9.4%)	7 (20.0%)	4 (12.5%)	7 (19.4%)
3	1 (3.1%)	0 (0.0%)	0 (0.0%)	0 (0.0%)	
Core features					
Fluctuating cognition	31 (96.9%)	34 (97.1%)	30 (93.8%)	35 (97.2%)	0.856
Visual hallucination	28 (87.5%)	28 (80.0%)	26 (81.3%)	29 (80.6%)	0.845
Parkinsonism	28 (87.5%)	31 (88.6%)	27 (84.4%)	29 (80.6%)	0.781
Hoehn & Yahr					
I	5 (15.6%)	5 (14.3%)	2 (6.3%)	5 (13.9%)	
II	7 (21.9%)	14 (40.0%)	12 (37.5%)	9 (25.0%)	
III	16 (50.0%)	12 (34.3%)	13 (40.6%)	15 (41.7%)	
IV, V	0 (0.0%)	0 (0.0%)	0 (0.0%)	0 (0.0%)	
Suggestive features	REM sleep behavior disorder	11 (34.4%)	17 (48.6%)	12 (37.5%)	12 (33.3%)	0.542
Severe neuroleptic sensitivity	3 (9.4%)	1 (2.9%)	0 (0.0%)	3 (8.3%)	0.261
Supportive features					
Repeated falls and syncope	8 (25.0%)	7 (20.0%)	6 (18.8%)	8 (22.2%)	0.933
Transient loss of consciousness	5 (15.6%)	2 (5.7%)	4 (12.5%)	0 (0.0%)	0.083
Severe autonomic dysfunction	7 (21.9%)	5 (14.3%)	7 (21.9%)	8 (22.2%)	0.809
Hallucinations in other modalities	10 (31.3%)	14 (40.0%)	12 (37.5%)	17 (47.2%)	0.600
Systematized delusion	9 (28.1%)	11 (31.4%)	11 (34.4%)	13 (36.1%)	0.905
Depression	11 (34.4%)	16 (45.7%)	9 (28.1%)	19 (52.8%)	0.160
Low occipital perfusion^a^	18 (85.7%)	19 (82.6%)	19 (82.6%)	18 (85.7%)	
Low MIBG uptake^a^	6 (100.0%)	7 (87.5%)	11 (91.7%)	7 (63.6%)	
Concomitant drugs					
L-dopa	5 (15.6%)	4 (11.4%)	9 (28.1%)	4 (11.1%)	0.202
Dopamine agonists	1 (3.1%)	1 (2.9%)	1 (3.1%)	2 (5.6%)	0.924
Antidepressants	2 (6.3%)	3 (8.6%)	1 (3.1%)	3 (8.3%)	0.796
Benzodiazepines	6 (18.8%)	13 (37.1%)	6 (18.8%)	9 (25.0%)	0.254

Data are mean (standard deviation) or number (%). ^a^Cerebral blood flow single photon emission computed tomography and MIBG myocardial scintigraphy were recommended but not mandatory. The former was available in 21, 23, 23, and 21 patients and the latter in 6, 8, 12, and 11 patients of the placebo, 3mg, 5mg, and 10mg groups, respectively. CDR = Clinical Dementia Rating; MIBG = ^123^I-metaiodobenzylguanidine; REM = rapid eye movement.

**Table 2:** Mean Changes in Clinical Variables from Baseline to Week 12 (Last Observation Carried Forward)

Variable	Group	Baseline			Change				
		Patients	Mean (SD)	*p*, ANOVA	Patients	Mean (SD)	Difference, 95% CI	*p*, *t* test a,b	*p*, ANCOVA^a^,^b^
MMSE	Placebo	32	18.3 (4.7)	0.271	31	-0.4 (2.7)			
	3mg	35	20.4 (4.1)	35	1.6 (3.8)	2.0 (0.4 to 3.7)	0.017	0.013
	5mg	32	19.8 (4.4)	32	3.4 (3.2)	3.8 (2.3 to 5.3)	<0.001	<0.001
	10mg	36	19.8 (4.4)	36	2.0 (3.3)	2.4 (0.9 to 3.9)	0.001	>0.001
WMS-R attention/concentration	Placebo	32	37.6 (11.3)	0.774	31	-0.9 (7.9)			
	3mg	35	37.9 (13.8)	34	3.1 (9.9)	4.1 (-0.4 to 8.5)	0.074	0.090
	5mg	32	40.5 (11.8)	32	5.6 (7.8)	6.5 (2.5 to 10.4)	0.001	0.002
	10mg	34	38.2 (12.7)	33	4.8 (7.4)	5.7 (1.9 to 9.5)	0.003	0.002
Category fluency	Placebo	32	7.2 (3.2)	0.170	31	0.3 (3.4)			
	3mg	35	8.2 (3.1)	34	1.2 (4.0)	0.9 (-1.0 to 2.7)	0.341	0.144
	5mg	32	8.6 (3.7)	32	1.6 (3.4)	1.3 (-0.5 to 3.0)	0.145	0.035
10mg	36	9.1 (4.1)	35	-0.5 (2.7)	-0.8 (-2.3 to 0.7)	0.286	0.905
Letter fluency	Placebo	32	10.7 (6.2)	0.445	31	0.3 (4.5)			
	3mg	35	10.2 (6.7)	34	1.1 (4.5)	0.8 ( -1.4 to 3.0)	0.474	0.461
	5mg	32	11.5 (6.1)	32	3.1 (5.8)	2.8 (0.1 to 5.4)	0.038	0.005
	10mg	36	12.6 (7.2)	35	1.7 (4.3)	1.4 (-0.7 to 3.6)	0.189	0.178
WAIS-III symbol digit modalities	Placebo	31	9.3 (8.2)	0.028	30	0.3 (5.9)			
	3mg	35	15.5 (11.4)	34	6.4 (7.9)	6.1 (2.6 to 9.7)	<0.001	<0.001
	5mg	32	15.9 (12.1)	32	6.9 (8.0)	6.6 (3.0 to 10.2)	<0.001	<0.001
	10mg	34	17.9 (14.8)	33	3.7 (7.9)	3.4 (-0.1 to 7.0)	0.057	0.021
VPTA form recognition	Placebo	32	3.6 (3.3)	0.252	31	-1.0 (2.9)			
	3mg	35	2.5 (3.6)	34	0.0 (2.7)	1.0 (-0.4 to 2.4)	0.145	0.167
	5mg	32	2.0 (2.4)	32	-1.1 (2.4)	0.0 (-1.4 to 1.3)	0.964	0.426
	10mg	35	2.7 (2.9)	34	-1.0 (2.1)	0.1 (-1.2 to 1.3)	0.921	0.568
NPI-10	Placebo	32	18.3 (8.9)	0.079	32	0.3 (17.5)			
	3mg	35	20.7 (12.8)	35	-3.9 (22.0)	-4.2 (-13.9 to 5.6)	0.396	0.602
	5mg	32	14.0 (8.3)	32	-5.5 (6.7)	-5.8 (-12.4 to 0.8)	0.086	0.047
	10mg	36	19.5 (12.8)	35	-8.0 (12.8)	-8.3 (-15.8 to -0.9)	0.029	0.019
NPI-2	Placebo	32	6.3 (4.0)	0.443	32	1.1 (5.7)			
	3mg	35	7.1 (4.1)	35	-2.1 (6.3)	-3.2 (-6.1 to -0.3)	0.032	0.025
	5mg	32	6.3 (4.8)	32	-3.3 (3.8)	-4.4 (-6.8 to -2.0)	<0.001	<0.001
	10mg	36	7.9 (5.4)	35	-4.6 (4.5)	-5.8 (-8.2 to -3.3)	<0.001	<0.001
NPI-4	Placebo	32	12.1 (6.3)	0.269	32	-0.3 (8.5)			
	3mg	35	11.5 (7.0)	35	-2.4 (10.8)	-2.1 (-6.9 to 2.6)	0.377	0.261
	5mg	32	9.0 (5.3)	32	-4.2 (4.9)	-3.9 (-7.3 to -0.4)	0.028	0.008
	10mg	36	11.9 (8.8)	35	-5.1 (7.4)	-4.8 (-8.7 to -1.0)	0.015	0.006
ZBI	Placebo	32	21.8 (10.1)	0.197	31	4.2 (10.4)			
	3mg	35	27.9 (13.9)	33	-1.3 (13.2)	-5.5 (-11.5 to 0.5)	0.069	0.301
	5mg	32	22.9 (11.5)	31	-0.7 (15.7)	-4.9 (-11.7 to 1.8)	0.149	0.172
	10mg	36	26.5 (16.1)	31	-5.0 (13.6)	-9.2 (-15.3 to -3.0)	0.004	0.035
UPDRS part III	Placebo	33	20.8 (10.6)	0.702	31	0.7 (3.8)			
	3mg	35	17.9 (9.0)	34	-0.5 (7.4)	-1.3 (-4.2 to 1.7)	0.393	0.397
	5mg	33	19.1 (10.7)	32	-0.5 (5.4)	-1.3 (-3.6 to 1.1)	0.281	0.358
	10mg	37	18.9 (11.6)	33	-1.0 (6.7)	-1.8 (-4.5 to 1.0)	0.200	0.258

The significant differences in the analyses using mixed-effect model for repeated measures were consistent with those based on last observation carried forward for MMSE, NPI-10, and NPI-2: MMSE (3mg, *p* = 0.010; 5mg, *p* < 0.001; 10mg, *p* = 0.003), NPI-10 (3mg, *p* = 0.115; 5mg, *p* = 0.160; 10mg, *p* = 0.028), NPI-2 (3mg, *p* = 0.003; 5mg, *p* < 0.001; 10mg, *p* < 0.001).

^a^Probability values are for the comparison between placebo and each active group.

^b^Significance level, *p* <0.0167 (= 0.05/3 with Bonferroni correction). ANCOVA = analysis of covariance; ANOVA = analysis of variance; CI = confidence interval; MMSE = Mini-Mental State Examination; NPI = Neuropsychiatric Inventory (NPI-10 = delusions - hallucinations - agitation/aggression - dysphoria - anxiety - euphoria - apathy - disinhibition - irritability/lability - aberrant motor behavior; NPI-2 = hallucinations - cognitive fluctuation; NPI-4 = delusions - hallucinations - dysphoria - apathy); SD = standard deviation; UPDRS =Unified Parkinson-s Disease Rating Scale; VPTA = Visual Perception Test for Agnosia; WAIS-III = Wechsler Adult Intelligence Scale; WMS-R = Wechsler Memory Scale-Revised; ZBI = Zarit Caregiver Burden Interview.

### Cognitive Function

**FIGURE 2:**
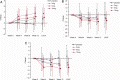
Mean changes from baseline in the (A) Mini-Mental State Examination and (B, C) Neuropsychiatric Inventory (B, NPI-10; C, NPI-2). Error bars represent standard deviation of the mean. LOCF = last observation carried forward.

Mean changes in MMSE scores were significantly higher at the final evaluation (LOCF) in the 5 and 10mg groups (5mg, 3.4, *p* < 0.001; 10mg, 2.0, *p* = 0.001) than in the placebo group (−0.4; see Table 2, Fig 2). When baseline values were adjusted as covariates, the difference between the 3mg and placebo groups was also significant. The results of the mixed-effect model analysis were consistent with those of LOCF analyses. The responder rate (MMSE change ≥3) was significantly higher in all donepezil groups (3mg, 42.9%, *p* = 0.013; 5mg, 65.6%, *p* < 0.001; 10mg, 44.4%, *p* = 0.007) compared to placebo (12.9%). No dose dependency was demonstrated on trend analysis. On the WMS-R attention/concentration and WAIS-III symbol digit tests, significant improvements were also noted in each dose group compared to placebo. No significant improvement was detected on the verbal fluency and visuoperceptual tests.

### Behavioral and Neuropsychiatric Symptoms

Scores for NPI-2 and NPI-4 were significantly more improved at the final evaluation (LOCF) in the 5mg (except NPI-4) and 10mg groups than in the placebo group (see Table 2, Fig 2). When baseline values were adjusted as covariates, the difference in NPI-4 between the 5mg and placebo groups was significant. The difference in NPI-10 between each active group and placebo did not reach the significance level. The results of the mixed-effect model analyses were consistent with those of LOCF analyses. The trend analyses demonstrated a linear dose-dependent improvement for NPI-2 (linear, *p* = 0.036; 5mg saturation, *p* = 0.076) but not for NPI-4 and NPI-10.

**FIGURE 3:**
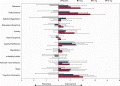
Mean changes (95% confidence intervals) of individual Neuropsychiatric Inventory items.

The NPI-plus domains Delusion, Hallucination, and Cognitive Fluctuation improved in all active groups, whereas they deteriorated in the placebo group (Fig 3). The differences between the placebo and both the 5 and 10mg groups were significant (5mg, *p* = 0.012, 0.014, and 0.004; 10mg, *p* = 0.002, <0.001, and <0.001 for each symptom, respectively).

### Global Function

The distributions of CIBIC-plus at the final evaluation (LOCF) in all active groups were significantly superior to that of placebo (*p* < 0.001 for each group; Table 3). The responder rates were 33.3%, 68.8%, 71.0%, and 64.3% in the placebo, 3mg, 5mg, and 10mg groups, respectively. The differences from placebo were significant in the 3 and 5mg groups (3mg, *p* = 0.010; 5mg, *p* = 0.004; 10mg, *p* = 0.034). No dose dependency was demonstrated on trend analysis.

**Table 3:** Distribution of the Clinician’s Interview-Based Impression of Change plus Caregiver Input at Week 12 (Last Observation Carried Forward)

Treatment Group	Total	Marked Improvement	Moderate Improvement	Minimal Improvement	No Change	Minimal Worsening	Moderate Worsening	Marked Worsening	Not Evaluable	*p*, Wilcoxon Rank Sum Test
Placebo	30	0 (0.0%)	1 (3.3%)	9 (30.0%)	5 (16.7%)	11 (36.7%)	4 (13.3%)	0 (0.0%)	0	
3mg	32	1 (3.1%)	6 (18.8%)	15 (46.9%)	8 (25.0%)	1 (3.1%)	0 (0.0%)	1 (3.1%)	0	<0.001
5mg	31	5 (16.1%)	7 (22.6%)	10 (32.3%)	4 (12.9%)	3 (9.7%)	2 (6.5%)	0 (0.0%)	0	<0.001
10mg	28	2 (7.1%)	3 (10.7%)	13 (46.4%)	9 (32.1%)	1 (3.6%)	0 (0.0%)	0 (0.0%)	1	<0.001

Percentages are based on the total number of evaluable patients in relevant treatment group. Probability values are for the comparison between placebo and each active group.

### Caregiver Burden

ZBI score was reduced significantly more in the 10mg group than in placebo at the final evaluation (LOCF; *p* = 0.004), although the difference did not reach the significance level after baseline value adjustment (see Table 2).

### Safety

AEs were reported in 71%, 69%, 82%, and 87%, respectively, of the placebo, 3mg, 5mg, and 10mg groups (Table 4). The majority were mild or moderate. The most common AE was elevated creatinine kinase (5.9%, 14.3%, 9.1%, and 13.5%, respectively). Cholinergic AEs such as diarrhea, nausea, anorexia, and abdominal discomfort were reported in some patients; however, no difference in incidence was noted between the placebo and any donepezil groups. Adverse parkinsonian events were reported in 2.9%, 8.6%, 12.1%, and 2.7%. The mean UPDRS part III score somewhat improved in all active groups at the final evaluation, whereas the score worsened in placebo, although the differences among groups did not reach the significance level (see Table 2). Adverse behavioral events were 11.8%, 22.9%, 15.2%, and 8.1% in the placebo, 3mg, 5mg, and 10mg groups, respectively; nevertheless, these differences were not statistically significant. The proportions of AEs leading to withdrawal were similar between groups: 11.8%, 8.6%, 3.0%, and 8.1%, respectively. Serious AEs occurred in 5.9%, 5.7%, 6.1%, and 10.8% of the respective groups. Of these, only 2 events, agitation in the placebo group and subarachnoid hemorrhage in the 3mg group, were judged to be related to the study drug. One serious AE in the 10mg group (worsening of hallucinations) occurred while the patient was still taking 3mg/day during the titration period. There were no clinically relevant differences in vital signs or electrocardiogram between the groups.

**Table 4:** Adverse Events

AEs	Placebo, n = 34	3mg, n = 35	5mg, n = 33	10mg, n = 37
Total	24 (70.6%)	24 (68.6%), *p* = 1.000	27 (81.8%), *p* = 0.391	32 (86.5%), *p* = 0.146
Severe AEs	2 (5.9%)	1 (2.9%)	0 (0.0%)	1 (2.7%)
Serious AEs	2 (5.9%)	2 (5.7%)	2 (6.1%)	4 (10.8%)
AEs leading to withdrawal	4 (11.8%)	3 (8.6%)	1 (3.0%)	3 (8.1%)
Gastrointestinal disorders	8 (23.5%)	1 (2.9%), *p* = 0.013	10 (30.3%), *p* = 0.589	13 (35.1%), *p* = 0.310	Anorexia	0 (0.0%)	1 (2.9%)	0 (0.0%)	2 (5.4%)
Diarrhea	4 (11.8%)	0 (0.0%)	4 (12.1%)	3 (8.1%)
Abdominal discomfort	1 (2.9%)	0 (0.0%)	2 (6.1%)	0 (0.0%)
Nausea	1 (2.9%)	0 (0.0%)	0 (0.0%)	2 (5.4%)
Vomiting	1 (2.9%)	0 (0.0%)	0 (0.0%)	1 (2.7%)
Parkinson symptoms	1 (2.9%)	3 (8.6%), *p* = 0.613	4 (12.1%), *p* = 0.197	1 (2.7%), *p* = 1.000
Psychiatric symptoms	4 (11.8%)	8 (22.9%), *p* = 0.341	5 (15.2%), *p* = 0.733	3 (8.1%), *p* = 0.702

Probability values are for the comparison between placebo and each active group.

### Discussion

In the present study, we found that donepezil improved both cognition and behavior in patients with DLB compared to placebo. Patients given 5 or 10mg donepezil showed greater improvement in the majority of the cognitive and behavioral measures, including the MMSE and NPI. Donepezil treatment also led to improved global function and reduced caregiver burden in this population. Because consistent improvements in many different measures across broad domains were observed, despite the exploratory nature of this study due to several limitations as discussed below, we believe that our findings demonstrated encouraging effects of donepezil for patients with DLB.

The majority of cognitive measures showed significant between-group differences. In particular, there was an apparent improvement in overall cognitive function, especially with the higher 2 doses; the mean changes in MMSE score favored donepezil by 2.0 to 3.8 points. This difference was larger than that reported in other studies of ChEIs in DLB, AD, and Parkinson disease with dementia (PDD).^15^, ^32^, ^33^ Improvement was also noted in the attentive-executive domains. We presume that a ceiling effect caused the nonsignificant outcome in the visuoperceptual domain.

Also noteworthy was the improvement of neuropsychiatric features and reduction of caregiver burden in the donepezil groups. The beneficial effect of donepezil was evident on each symptom domain characteristic of DLB (delusion, hallucination, and cognitive fluctuation), as generally consistent with the previous rivastigmine study^15^ except for apathy. For NPI-2, a linear dose-response relationship was demonstrated. Caregiver burden also was reduced significantly at the highest dose, 10mg/day.

Patients who received donepezil also demonstrated improved global function, as measured by CIBIC-plus. A higher percentage of patients showed improvement, and fewer patients worsened in each donepezil group than in placebo. The beneficial effect seemed greater than those of ChEIs reported for patients with AD and PDD.^33-35^ In a trial of rivastigmine for PDD, improvement of activities of daily living, which would reflect treatment-induced changes in cognitive, behavioral, and motor symptoms, was reported.^33^ Such an outcome may also be useful to compare the clinically meaningful impacts of the treatment among trials.

AEs were not rare; however, only approximately 8% of the study population withdrew due to AEs, and the prevalence of withdrawal or AEs, including typical cholinergic side effects, did not differ among treatment groups. Although symptoms of parkinsonism were reported as AEs somewhat more frequently in the 3 and 5mg groups than in the placebo group, the difference was not reflected in the mean UPDRS part III score. Indeed, the score demonstrated numerical, although nonsignificant, improvement in the highest dose group. Cholinergic treatment theoretically exacerbates parkinsonism. However, the possible beneficial effects of donepezil observed in this study suggest that the use of ChEIs should not necessarily be avoided in the treatment of DLB due to concern of possible parkinsonism. These unexpected effects, despite not being confirmed, might be explained by a complicated neuronal network for motor control.

As the discontinuation rate was relatively low, and there was no significant difference among groups, it is unlikely that exclusion bias caused by early termination affected the efficacy results. Both the LOCF analysis and the mixed-effect model analysis consistently showed favorable results.

As an aim of this study was to explore targetable clinical presentations of DLB, we did not set a specific primary endpoint despite assigning multiple efficacy outcome measures, which could be a major limitation. In addition, cognitive fluctuation was measured by an unestablished tool, which is well equipped but not yet validated. Another limitation is that nearly half of the centers enrolled only 1 or 2 patients, which may have caused an inter-rater discordance of the clinical ratings, although a training and certification course was mandatory for the investigators. Also, the small sample, short duration of treatment, and lack of formal dose-response comparison are evident limitations. Nevertheless, the results of this study strongly suggest that donepezil is safe in patients with DLB, and provide a preliminary indication of its clinical effectiveness in terms of cognitive function, behavioral symptoms, and global function of DLB, and consequently in effecting a reduction of caregiver burden. The findings of the present study with donepezil should be verified in a confirmatory clinical trial. In addition, long-term effects should be examined. Although both 5mg/day and 10mg/day seemed to be beneficial, 10mg/day was somewhat more beneficial in terms of behavioral symptoms. The optimum dose should be determined in a follow-up trial, in which dose titration with patients unable to tolerate 10mg/day being allowed to take 5mg/day would be a sensible design.

## Appendix Donepezil-DLB Study Investigators

Eizo Iseki (Juntendo Tokyo Koto Geriatric Medical Center), Sadao Katayama (Hiroshima-nishi Medical Center), Yasuto Higashi (Himeji Central Hospital), Mamoru Hashimoto (Kumamoto University Hospital), Tatsuo Yamada (Fukuoka University Hospital), Takemi Kimura (Kikuti National Hospital), Yoko Nakano (Sukoyaka-silver Hospital), Satoshi Orimo (Kanto Central Hospital), Aki Nakanishi (Osaka City Kosaiin Hospital), Yuichi Maruki (Saitama Neuropsychiatric Institute), Tadashi Tsukamoto (National Center of Neurology and Psychiatry), Aoi Yoshiiwa (Oita University Hospital), Tomonobu Kato (Osaka Red Cross Hospital), Yoshiyuki Nishio (Tohoku University Hospital), Noriyuki Matsukawa (Nagoya City University Hospital), Masanori Hiji (Vi-hara Hananosato Hospital), Masayuki Yokochi (Ebara Hospital), Norio Taniguchi (Asakayama General Hospital), Koichi Mizoguchi (Shizuoka Institute of Epilepsy and Neurological), Miyuki Kobayashi (Komoro Kogen Hospital), Haruo Hanyu (Tokyo Medical University Hospital), Tatsuru Kitamura (Takamatsu Hospital), Yasuhiro Tsugu (Toyokawa City Hospital), Koichi Okamoto (Gunma University Hospital), Yuri Kitamura (Nanohana Clinic), Kenichi Shimada (Hyogo Brain and Heart Center), Yasumasa Yoshiyama (Chiba-East Hospital), Satoshi Takahashi (Iwate Medical University Hospital), Kazuo Shigematsu (Minami Kyoto Hospital), Hiroaki Kazui (Osaka University Hospital), Masahiro Akishita (Tokyo University Hospital), Takashi Kanda (Yamaguchi University Hospital), Yasuji Yamamoto (Kobe University Hospital), Yasuhiro Kawase (Kawase Neurology Clinic), Yukihiko Washimi (National Center for Geriatrics and Gerontology), Yasushi Osaki (Kochi University Hospital), Hiroaki Hino (Yokohama Hoyu Hospital), Toshimasa Matsuoka (Kurume University Hospital), Fukashi Udaka (Sumitomo Hospital), Toshifumi Kishimoto (Nara Medical University Hospital), Hiroaki Oguro (Shimane University Hospital), Hideyuki Sawada (Utano Hospital), Naoki Fujii (Omuta Hospital), Takashi Asada (Tsukuba University Hospital), Hiromori Takeuchi (Saigata Hospital), Takamasa Okayama (Osaka Police Hospital), Junya Sugawara (Akita University Hospital), Koichi Mino (Kobe City Medical Center West Hospital).
